# Professor Johannes Vester, President of the Academy for Multidisciplinary Neurotraumatology (AMN): Adapted Interview from the 21^st^ AMN Congress in Vienna, Austria

**DOI:** 10.25122/jml-2024-1011

**Published:** 2024-07

**Authors:** Stefana-Andrada Dobran, Alexandra Gherman

**Affiliations:** 1 RoNeuro Institute for Neurological Research and Diagnostic, Cluj-Napoca, Romania; 2 Sociology Department, Babes-Bolyai University, Cluj-Napoca, Romania

**Interviewee: Professor Johannes Vester**

**Interviewer: Stefana-Andrada Dobran**

Professor Johannes Vester has served as the President of the Academy for Multidisciplinary Neurotraumatology (AMN) since 2018. He has been the Head of Biometry & Clinical Research at the Institute for Data Analysis and Study Planning (IDV) in Germany since 2018 and Invited Associate Professor at the Department of Neurosciences at Iuliu Hatieganu University of Medicine and Pharmacy, Cluj-Napoca, Romania, since 2017.

With a background in medicine, Professor Vester researched pattern recognition in the visual brain and developed a pharmacodynamic Neuron Simulation Model at the Institute for Medical Documentation and Statistics (University of Cologne). He has conducted over 100 training courses on biometry for clinical research professionals and taught at various universities and international institutions. Throughout his career, Professor Vester has planned and evaluated around 150 randomized clinical studies worldwide.

He is a member of several international Advisory Boards and Steering Committees and has contributed as a biometric expert in regulatory authority panels, including hearings with the United States Food and Drug Administration (FDA), the European Medicines Agency (EMA), and Germany’s Federal Institute for Drugs and Medical Devices (BfArM). He is also involved in workshops for the International Biometric Society (IBS) and serves as a statistical peer review member for leading medical journals.

Professor Johannes Vester holds key roles in several organizations, serving as the Statistical Expert and Elected Member of the International Scientific Committee for the Society for the Study of Neuroprotection and Neuroplasticity (SSNN), and Co-Chair of the EAN Guideline Task Force on Neurorehabilitation.

**S.D.: Dear Professor Vester, as we are facing the horizon of your fifth year of AMN presidency, please share with us some of your past and future thoughts regarding the evolution of the Academy**.

J.V.: Yes, evolution is the right word! You know, science and medical knowledge are always a snapshot; it can never capture the full truth. It’s an ongoing process, and this applies also to scientific societies, as the AMN: it has to advance in order to stay a living entity. So, I’m excited that important developments of the AMN have come to life in recent years, also during my presidency here: great educational endeavors, production of advanced international treatment guidelines, cutting-edge research implementing the multidimensional approach – it was so necessary to promote this. It’s a living society, and I’m excited to be a part of this. And, in the end, this is also reflected by the change in the dynamic way the Academy outreaches the people, which, last but not least, is also expressed in the rebranding of the AMN.

**S.D.: When looking at the development of translational and clinical research concepts in the last decade, one might see dramatic changes. How would you outline the new multidimensional methodology applied to classic evidence-based medicine concepts?**

J.V.: Good question. In fact, there are dramatic changes. If we have a look at TBI (traumatic brain injury) research, the Glasgow Outcome Scale, for instance — a single-functional assessment scale — was dominating clinical research for decades. With only a few steps between death and full recovery. With a very low resolution of outcome, mostly even further broken down to simple binary scale of just favorable or unfavorable outcomes. Such a simple black-and-white thinking, which dominated research also in other fields like stroke, as with the modified Rankin Scale. Of course, that can never capture the full breadth of outcomes in such complex domains as recovery after traumatic brain injury. Such simplified approaches, which dominated research for so long, are just not able to identify important deficits of the patient. That’s where we have to focus – on the patient as a human being. As opposed, the multidimensional approach addresses the full breadth of consequences after neurotrauma, such as physical functioning, but also cognitive functioning, mental health, social integration, social communication, quality of life – that’s also important – that I feel after the trauma coming back to what I was before, not just that I can move my hand; also including neuropsychiatric, cognitive, emotional, psychosocial aspects – very important! This way, I would say it’s a holistic approach across all pertinent health states and life domains of a patient. It’s *patient-centered*, considering multiple facets of their health and well-being. And, fortunately, cutting-edge statistical procedures, which are necessary based on correlation-sensitive full-scale analyses, are meanwhile established for this approach. The International Biometric Society, for instance, initiated workshops on how to apply these high-tech new statistical procedures. The AMN was an integrative part of these important developments, disseminating the necessary knowledge to the people, to the community, and consistently promoting the importance of the switch from the old single-outcome paradigm – black and white – to future-oriented multiple-outcome approaches which capture the full picture of the patient as a human being.


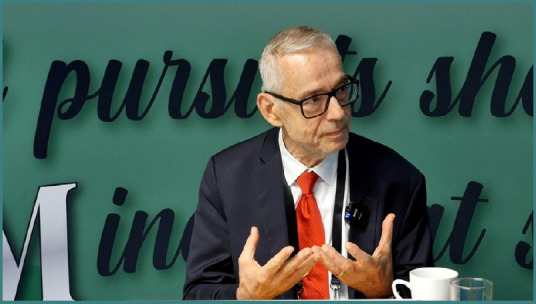


**S.D.: In the field of TBI, how do you see the contribution of non-interventional studies and real-world evidence to the general concept of evidence-based medicine?**

J.V: Well, let’s say, basically real-world evidence is an important contributor to the knowledge of how best to treat patients. *Why?* Because at the end, it’s the real world where the benefit has to happen, with the real patient. And, unfortunately, non-interventional trials, when it comes to international guideline production, they enter the stage through the door of low evidence: whatever they are, the first label is ‘low evidence’. Randomized controlled clinical trials enter the stage with high evidence. Unfortunately, those non-interventional trials, if they have dropouts, if they have some bias problems, they are further downgraded to very low evidence or no evidence. That’s a tragedy because, if observational trials are downgraded that way, years of research are lost completely, and you have nothing at the end in your hand. Again and again, that happened in the last 10, 20, 30, 40 years. Fortunately, new effective pathways are now available and have to be established to avoid this tragedy.

And one of the key milestones to avoid this was the GRACE guideline: that is the guideline for high-quality effectiveness research in observational trials or non-interventional trials with a very strict comprehensive ensemble of rules and to-dos, now paving the way for non-interventional trials to be upgraded to higher levels of evidence. Such trials following these new high-quality principles can now become serious co-players in the world of evidence-based medicine. So, I’m not the poor kid anymore: you have to go to the low evidence. If you come with this high-quality approach during the whole conduct of the trial then you can contribute in terms of evidence and the years of research are not lost. That’s an exciting development.


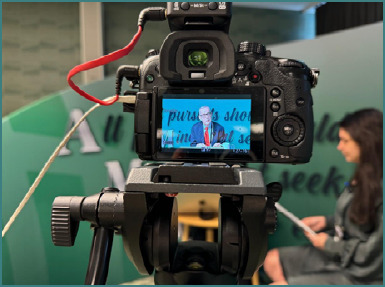


**S.D.: To your knowledge, are there specifical medical journals keen to publish real-world evidence studies and is there a particular trend in this respect?**

J.V.: I would say that we want to play on the international level of evidence-based medicine. We should treat such high-quality non-interventional trials equally, and try to publish in the same journals as randomized trials. You know why? Because otherwise we have the danger that we put [them] into a niche and that these trials are separated. This is the opposite of what we want. We want to get them from the seller on the table because they are now following these high-quality principles, so they are comparable to randomized trials and they can speak in terms of evidence-based medicine. So, I would not separate that, I would not go to specialized journals just for this kind of trials.

**S.D.: And for my last question, could you please describe some advanced projects that illustrate the development of the AMN within the new frame of thinking?**

J.V.: Yes, let’s start with the CAPTAIN series of trials: that was the biggest breakthrough and, I would say, the most important development for the future. It was the first multidimensional approach in TBI research based on full scales, applying all these new cutting-edge methodologies, and, in fact, the CAPTAIN trials, which are completed now, are the worldwide first randomized controlled clinical trials in TBI based on such a true multidimensional approach — and it was a great endeavor. If you do something new you will see that there is a lot of resistance; people don’t want to leave what they did since 20, 30, 40 years, but it was successful in the end and the methodology, because it was so new, was published in advance, very important, in the Journal of Neurotrauma. And after a challenging road of more than six years, I would say, the CAPTAIN trials did not only demonstrate the feasibility of such an approach, which is very important, but much more, leading to high evidence in terms of consistent, statistically significant results. So, we have something at hand: we have evidence at hand, more evidence, more test power than we would have with an arbitrary selection of single-outcome scales, with this multidimensional approach – It’s like an airplane which you are flying with more than one motor: it’s much more stable and it has more power – and so it was a very successful but very challenging road to establish that, and after the first two successful CAPTAIN trials in the series, the third one is now underway. The C-RETURN study follows this approach, applying high-quality measures, not only in the planning but also in the conduct of this trial with high-quality and risk-based management of the data and the whole conduct. So, then to mention the CREST study: that’s, let’s say, the real-world translation of the CAPTAIN series, which was randomized, controlled, and double-blind. The CREST is real-world but with the same multidimensional approach, because it’s also important to show that we can work with this approach, that we have power, test power, and that we can create evidence for our high-quality comparative effectiveness as expressed by these new GRACE guidelines. And, last but not least, I should mention the performance of formal meta-analyses combining the existing evidence of these multidimensional approaches by state-of-the-art methodology based on principles like Cochrane, Consort, PRISMA guidelines, and so on. That also was successful, and I should mention the PRESENT (Patient REgistry – Short Essential NeuroTrauma) Project - which is somehow related to the CREST real-world approach, collecting the necessary data items within the framework of a registry and thus allowing the inclusion of a greater range of population and a greater range of real-world settings. So this is ongoing in the moment, and all these are cutting-edge approaches, opening new pathways to the future, and that is why the AMN is here, at the end, for the benefit of future patients.


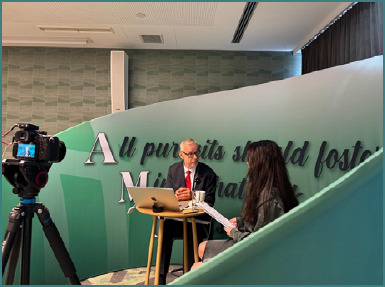


**S.D.: Thank you so much for the interview!**

J.V: You’re welcome. Thank you! Thank you very much for the questions.

